# Post-COVID-19 lichen planus annularis: report of a rare association^⋆^

**DOI:** 10.1016/j.abd.2021.10.016

**Published:** 2023-01-25

**Authors:** Laura Murari Mondadori, Helena Barbosa Lugão, Fernanda André Martins Cruz Perecin, Marco Andrey Cipriani Frade

**Affiliations:** Hospital das Clínicas, Ribeirão Preto Faculty of Medicine, Universidade de São Paulo, Ribeirão Preto, SP, Brazil

Dear Editor,

Since the beginning of the new coronavirus (SARS-CoV-2) pandemic, several cases of extrapulmonary involvement have been reported, including cardiovascular, gastrointestinal, neurological, and cutaneous manifestations. A wide variety of dermatological conditions related to COVID-19 infection have been reported;^1^, ^2^ however, reports of lichen planus (LP) associated with COVID-19 are scarce in the literature.^3^, ^4^

A 56-year-old male patient complained of pruritic lesions that appeared on his lower limbs six months before. He mentioned that the lesions appeared approximately one week after the onset of COVID-19 infection symptoms, which was confirmed by RT-PCR. The patient had a mild respiratory clinical picture, without the need for hospitalization, and used ivermectin and hydroxychloroquine, prescribed at the service where he was originally treated. After the rash appeared, he used oral fluconazole and topical ketoconazole, with no improvement. The patient had a history of HIV infection, with an undetectable viral load for several years, without other comorbidities and with negative serology for syphilis, hepatitis B, and hepatitis C. He was undergoing regular treatment with lamivudine (3TC), tenofovir disoproxil fumarate (TDF) and dolutegravir (DTG), without recent changes in medications.

On dermatological examination, the lesions were clinically and dermoscopically compatible with LP (Figure 1, Figure 2). He had no ungueal or oral mucosa lesions. Biopsies of two lesions were performed (Fig. 3), confirming the diagnosis of LP annularis after COVID-19 infection.

**Figure 1:**
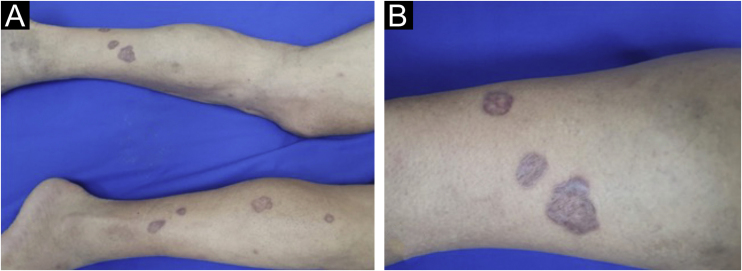
(A) Violaceous lesions, with raised edges and an atrophic center, with a shiny, lichenoid surface on the lower limbs, ranging in size from 0.5 to 2 cm. (B) Lichenoid lesion in greater detail, showing its annular aspect.

**Figure 2:**
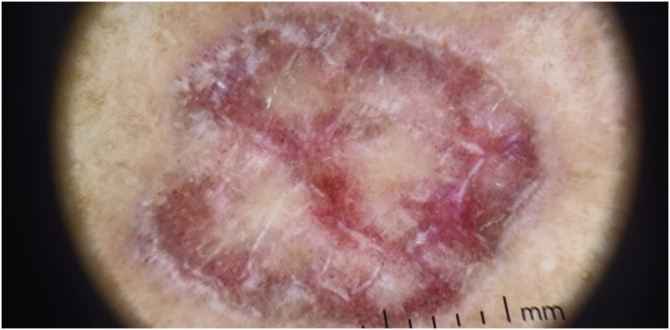
Dermoscopy of lesion on the right lower limb, showing linear Wickham striae.

**Figure 3:**
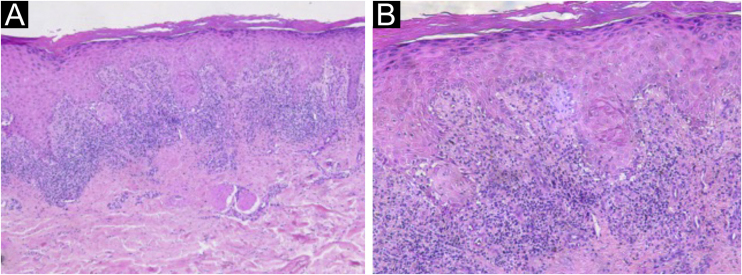
Histopathology of the right lower limb lesion. (A) Epidermis showing compact hyperkeratosis, parakeratosis, hypergranulosis, mild irregular acanthosis, mild spongiosis and superficial dermis with perivascular and periadnexal lymphohistiocytic lichenoid inflammatory infiltrate in a band-like disposition, without alterations in the deep dermis (Hematoxylin & eosin, ×50). (B) Higher magnification showing basal vacuolar changes, a subepidermal cleft and rare apoptotic keratinocytes (Hematoxylin & eosin, ×100).

Lichen planus is an immune-mediated dermatosis of unknown cause, which affects less than 1% of the population, mainly middle-aged adults, and may affect the skin, hair, nails, and mucous membranes.^5^ Association with hepatitis C, other viral infections, vaccines, and autoimmune diseases such as vitiligo, dermatitis herpetiformis, and pemphigus has been reported.^5^ The annularis form is considered a rare variant of the LP. Although several skin manifestations have been associated with COVID-19, few cases of post-COVID-19 LP have been reported.^3^, ^4^

A possible association between HIV infection and LP has already been reported. In the present case, we consider that the LP was triggered by the COVID-19 infection, since the patient had been diagnosed with HIV 24 years before, without changes in the medications of chronic use, with the appearance of lichenoid lesions timely associated with COVID-19. SARS-CoV-2 infection can stimulate cytotoxicity by TCD8+ lymphocytes and Th17 cells, changes that also participate in the pathogenesis of LP, and this can persist even after the resolution of the triggering viral infection.^4^ Moreover, we do not consider LP as being triggered by the medications used during the coronavirus infection, since no reports of LP triggered by ivermectin or hydroxychloroquine use have been identified in a literature review, as well as because of the persistence of the lesions after the discontinuation of these drugs.

We report a case of annular LP that appeared one week after infection with COVID-19, suggesting an association between the diseases. There is a scarcity of reports of lichenoid lesions associated with COVID-19, with only one other report of the rare LP annularis variant triggered by SARS-CoV-2 infection having been identified.^3^, ^4^

## Financial support

None declared.

## Authors’ contributions

Laura Murari Mondadori: Design and planning of the study; data collection, or data analysis and interpretation; statistical analysis; drafting and editing of the manuscript or critical review of important intellectual content; collection, analysis, and interpretation of data; effective participation in research orientation; intellectual participation in the propaedeutic and/or therapeutic conduct of the studied cases; critical review of the literature.

Helena Barbosa Lugão: Design and planning of the study; data collection, or data analysis and interpretation; statistical analysis; drafting and editing of the manuscript or critical review of important intellectual content; collection, analysis, and interpretation of data; effective participation in research orientation; intellectual participation in the propaedeutic and/or therapeutic conduct of the studied cases; critical review of the literature; approval of the final version of the manuscript.

Fernanda André Martins Cruz Perecin: Design and planning of the study; data collection, or data analysis and interpretation; statistical analysis; drafting and editing of the manuscript or critical review of important intellectual content; collection, analysis, and interpretation of data; effective participation in research orientation; intellectual participation in the propaedeutic and/or therapeutic conduct of the studied cases; critical review of the literature; approval of the final version of the manuscript.

Marco Andrey Cipriani Frade: Design and planning of the study; data collection, or data analysis and interpretation; drafting and editing of the manuscript or critical review of important intellectual content; collection, analysis, and interpretation of data; effective participation in research orientation; intellectual participation in the propaedeutic and/or therapeutic conduct of the studied cases; critical review of the literature; approval of the final version of the manuscript.

## Conflicts of interest

None declared.
